# Editorial: Exploring AI’s role in disease prediction and diagnosis through medical big data

**DOI:** 10.3389/fmolb.2026.2005303

**Published:** 2026-10-26

**Authors:** Yulin Zhang

**Affiliations:** College of Mathematics and Systems Science, Shandong University of Science and Technology, Qingdao, China

**Keywords:** artificial intelligence, biomarker, machine learning, predictive modeling, interpretability

Modern translational medicine increasingly leverages multi-omics data and artificial intelligence to uncover disease biomarkers and build clinical prediction tools. Nevertheless, conventional statistical methods and “black-box” machine learning models often impede biological interpretability and real-world clinical adoption. The four studies in this Research Topic present distinct yet complementary workflows covering metabolomics, transcriptomics, population health surveys and electronic health record-based clinical trial design. Each study combines custom-tailored datasets, well-designed analytical pipelines, and (in most cases) explainable artificial intelligence (XAI) to address unmet clinical needs for genetic syndromes, cardiovascular disease, colorectal pre-malignant lesions and severe influenza.

The first article investigates plasma metabolomic biomarkers for Down syndrome (trisomy 21, T21). Colak et al. aimed to identify pathway-specific metabolic biomarkers that distinguish individuals with Down syndrome from healthy controls. Using plasma LC-MS metabolomics data from 316 participants with T21 and 103 control participants, five ensemble machine learning classifiers (AdaBoost, LightGBM, Random Forest, KTBoost, XGBoost) were compared, and KTBoost delivered the best classification performance. SHAP-based XAI analysis identified L-Citrulline, Kynurenin, Prostaglandin A2/B2/J2, Urate and Pantothenate as the top biomarker candidates, which reflect oxidative stress, mitochondrial dysfunction and inflammatory dysregulation in T21.

The second paper presents a multi-center clinical trial protocol for deep learning-based early detection of severe influenza. Chen and Bo designed a five-phase, multi-center, double-blinded clinical trial to validate a deep learning model for early severe influenza detection using electronic health record data from 87 hospitals in Jiangsu, China. The study hypothesizes that AI-assisted diagnosis will improve the AUC by more than 0.15 and reduce misdiagnosis rate by at least 30% compared with physician-only assessment, and maintain consistent performance across subgroups defined by age, comorbidity and hospital resource level.

The third study introduces a stacking-ensemble interpretable prediction model for heart-disease risk. Chen et al. constructed a two-level stacking-ensemble classifier that integrates Naive Bayes, Decision Tree, CatBoost and Gradient Boosting Tree to predict heart disease status using large-scale population health survey data. The stacking model outperformed single base learners, reaching 86.69% accuracy and an AUC-ROC of 0.97. Global and local SHAP interpretation identified sleep duration, self-reported general health status, age category and BMI as the dominant cardiovascular risk factors, and characterized feature interaction patterns such as the joint effect of sleep and health status.

The fourth work focuses on biomarker discovery for the malignant transformation of colorectal polyps. He et al. combined bioinformatics and machine learning to screen for gene markers associated with the transition from colorectal polyps to colorectal cancer. By integrating bulk RNA-seq, single-cell RNA-seq and three feature selection algorithms (Boruta, LASSO regression, XGBoost), six core genes (EIF2S3, GTF3A, HMGA1, HSP90AB1, PABPC1, S100A11) were selected to build a ridge regression diagnostic model. The model achieved strong performance in both internal and external validation cohorts; qRT-PCR confirmed the upregulated expression of these genes in CRC cell lines, and survival analysis linked high expression of EIF2S3 and S100A11 to poorer patient prognosis.

Across all four studies, the value of interpretability and multi-source validation is clear. Metabolomics-oriented XAI prioritizes plasma biomarker candidates for genetic diseases; stacking ensemble combined with SHAP provides person-level risk explanation for population-derived epidemiological data; multi-algorithm transcriptomic filtering narrows down gene signatures for pre-cancerous lesions; multi-phase prospective trial frameworks translate retrospective AI models into clinical practice. Common limitations across the studies include a lack of large-scale prospective external validation, confounding clinical variables, and the challenge of distinguishing correlation from causation. Future research should prioritize multi-center prospective cohorts, harmonized multi-omics integration, and intervention-oriented studies to move biomarker candidates toward clinical application. This Research Topic shows that neither high-accuracy machine learning nor omics profiling alone is sufficient; interpretability and rigorous clinical validation are the core pillars of translating AI-augmented precision medicine into practice.

